# Early vaccination of laying hens with the live bivalent *Salmonella* vaccine AviPro™ Salmonella DUO results in successful vaccine uptake and increased gut colonization

**DOI:** 10.3389/fmicb.2023.1327739

**Published:** 2024-01-15

**Authors:** Shaun A. Cawthraw, Adam Goddard, Tom Huby, Isaac Ring, Louise Chiverton, Doris Mueller-Doblies

**Affiliations:** ^1^Department of Bacteriology, Animal and Plant Health Agency (APHA - Weybridge), New Haw, Surrey, United Kingdom; ^2^Elanco Animal Health, Form 2, Bartley Way, Bartley Wood Business Park, Hook, United Kingdom; ^3^Elanco Austria GmbH, Quartier Belvedere Central, Vienna, Austria

**Keywords:** *Salmonella* Enteritidis, *Salmonella* Typhimurium, live vaccines, laying hens, vaccination

## Abstract

**Introduction:**

*Salmonella* Enteritidis and *S.* Typhimurium are the two most clinically important zoonotic *Salmonella* serovars and vaccination of breeding and laying hens affords effective *Salmonella* control. The use of live vaccines has proven beneficial for a number of reasons, including ease of application, protection from the first day of life onwards and initiation of a strong local immune response. Live vaccines can be applied in the drinking water from the first day of life onwards, but some rearers choose to wait until the end of the first week to ensure sufficient water consumption. However, this practice leaves the birds unprotected during the crucial first week of life, where they are most susceptible to colonization by field strains. The aim of this study was to determine if successful vaccine uptake is achieved when layer pullets are vaccinated as early as day one.

**Methods:**

Three pullet flocks were vaccinated at 1, 2, 3 or 5 days-of-age with AviPro™ Salmonella DUO, a live vaccine containing attenuated strains of *S.* Enteritidis and *S.* Typhimurium (Elanco Animal Health, Cuxhaven, Germany). The vaccine was administered via the drinking water following manufacturer’s instructions. Two days post-vaccination, 10 birds per flock were culled and caecal and liver samples taken, along with two pools of faeces per flock. Levels of vaccine strains were determined by quantitative and qualitative bacteriology.

**Results:**

Vaccine strains were detected in all birds from all age groups indicating successful uptake of the vaccine. Levels of the *S.* Enteritidis vaccine were higher than levels of the *S.* Typhimurium vaccine, with the latter frequently only detectable following enrichment. There was an inverse correlation between age and caecal levels of vaccines, with the highest numbers seen in birds vaccinated at 1-day-of-age. Interestingly, *S.* Enteritidis vaccine strain levels in liver samples were highest when birds were vaccinated at 5 days-of-age.

**Discussion:**

These results show that successful uptake of both vaccine strains was evident in all age groups. The earlier the chicks were vaccinated, the higher the vaccine levels in caecal contents. We therefore recommend vaccination of pullets as early as practicably possible to ensure protection against exposure to field strains.

## Introduction

Zoonotic strains of *Salmonella enterica* are some of the most important food-borne pathogens worldwide, causing an estimated 78.7 million illnesses and 59,000 deaths each year (World Health Organization, 2015). More than 2,600 serovars are known, most of which are a public health concern. Although *Salmonella* can be present in a variety of foods of animal and non-animal origin, European data show that the majority of *Salmonella* outbreaks are linked to the poultry sector, in particular to eggs and egg products (European Food Safety Authority, 2022).

The serovar responsible for most egg-related human illness and outbreaks is *S.* Enteritidis, which started to emerge in Europe and in the US in the 1980s and has been the most important serovar in many parts of the world since (O’Brien, 2013; Lane et al., 2014; World Health Organization, 2018). Although significant progress has been made in the control of *S.* Enteritidis in the laying hen sector in Europe, this particular serovar is still causing the highest number of outbreaks and outbreak-related illnesses, most of which can be linked to eggs and egg products (European Food Safety Authority, 2022).

Control of zoonotic *Salmonella* in the poultry sector can only be achieved using a holistic approach, including high biosecurity, adequate pest control and good management practices. An additional helpful tool in the battle against *Salmonella* infection are vaccines, and to date, vaccines offering protection against *S.* Enteritidis and *S.* Typhimurium are widely used in many parts of the world.

Inactivated *Salmonella* vaccines, which are administered through intramuscular injection, first became available in the early 1990s (Lane et al., 2014) and were subsequently used mainly in the breeding sector. However, because of the inconvenience of application, their use in laying hens was limited. Only the introduction of live attenuated vaccines a few years later and their extended use in both the breeding and laying hen sectors led to a significant reduction in human case numbers and laying hen prevalence (Lane et al., 2014). The use of live vaccines proved particularly efficient as they can be applied via drinking water and as early as on the first day of life.

It is acknowledged that live *Salmonella* vaccines generally confer better protection than killed vaccines, because they stimulate both cell-mediated and humoral immunity, including local, mucosal IgA responses (Zhang-Barber et al., 1999; Van Immerseel et al., 2005). When live vaccines are administered orally to young birds (through drinking water application or hatchery spray), they lead to extensive gut colonization and a strong local immune stimulus. Early colonization of newly-hatched chicks with a live vaccine strain leads to the so-called colonization-inhibition effect which prevents the colonization of the gut with other bacteria, thus offering an early protection which goes even beyond the vaccine-specific serovar (Van Immerseel et al., 2005).

Ideally, pullets should be vaccinated as early as possible to protect them during the first weeks of life when they are most susceptible to colonization by *Salmonella* (Shivaprasad et al., 2013). However, the main reason why some pullet-rearers often wait until the end of the first week before administering the first dose is the worry that the water-intake of the pullets may be too low during the first few days, potentially leading to a reduced uptake of the vaccine.

The efficacy of the vaccine used in this trial has been proven over many years ago, both through controlled trials and from experience in the field since the early 1990s. Several studies showed successful protection of birds with the first dose applied at day one (Gantois et al., 2006; Eekhout et al., 2018; Huberman et al., 2019). However, in these trials, the vaccine was administered via oral gavage to ensure the successful vaccination of each individual chick. In commercial poultry production, it is not possible to administer a live vaccine in such a way to individual birds, so application via the drinking water line has been a well-established method for vaccine administration. As there are sufficient data to show that the vaccine is efficacious when applied at an early age, we focused on the question of successful uptake.

The aim of this field study was therefore to analyze and compare the vaccine uptake of pullets which received their first dose of the live, bivalent *Salmonella* vaccine AviPro™ Salmonella DUO (Elanco Animal Health) at 1-, 2, 3- or 5-days-old (d.o.). The results of this study may help pullet rearers in their decision-making process on when to administer the first dose safely and effectively, ensuring that the birds receive an appropriate dose of the vaccinal product in a timely manner.

## Materials and methods

### On-farm vaccination

Four commercial pullet rearing farms in England with at least three houses each were recruited for the study. Chicks were sourced from a reputable UK hatchery and had been vaccinated against Marek’s disease, coccidiosis and Infectious Bronchitis at the hatchery according to standard vaccination protocols. No *Salmonella* was detected in routine National Control Program samples taken from the flocks used in the study. On farm, they were vaccinated with AviPro™ Salmonella DUO, a licensed vaccine consisting of two attenuated live strains: *S.* Enteritidis strain Sm24/Rif12/Ssq and *S.* Typhimurium strain Nal2/Rif9/Rtt (vSE and vST, respectively). Chicks were vaccinated either at 1-, 2-, 3- or 5-days-old. Vaccines were prepared and administered on farm under the supervision of qualified personnel from the vaccine company. Briefly, freeze-dried vaccine was reconstituted according to manufacturer’s instructions using mains water and a water stabilizer (Aviblue, Lohmann Animal Health, Maine, USA). The vaccine was administered via a water proportioner, dosing at 2%. Once the vaccine was reconstituted in stabilized water, the nipple lines were lifted and the lines were primed with vaccine, removing all clear surplus water until the blue dye was present at the end of each nipple line. Once all the nipple lines had been primed, they were lowered to allow the chicks to drink. The early vaccination in the first days of life has always been challenging as the water system holds more water than the chicks would drink over a normal vaccination period of up to 3 h. Key is knowing the capacity of the system (total volume of water) to enable successful vaccination to take place in the first days of life. Vaccines were administered for up to 12 h with the following rates dependant on the birds’ age: day one—2.5 ml per chick, day two—4 ml per chick, day three—4.5 ml per chick, day 5—5.5 ml per chick.

### Sampling

For each farm on the day of vaccination, vaccine/drinking water samples were taken from two points on the drinker line. Two days post-vaccination, 10 chicks were randomly selected and euthanised and two pools of 10 individual fecal droppings were collected from each flock. Samples and carcases were despatched in chilled containers to the APHA laboratory on the day of collection and processed the same day.

### Bacteriology

Upon receipt at APHA, samples of caecal contents and liver tissue were taken from each carcase. Samples were weighed and homogenized in Buffered Peptone Water (1:9, w:v) supplemented with rifampicin (100 μg/ml; Merck, Germany). Homogenized samples and vaccine samples were serially diluted (1/10) in PBS and dilutions spread-plated onto selective agars (200 μl for the starting homogenate, and 100 μl for the subsequent dilutions). For detection of vSE, brilliant green agar (BGA) supplemented with rifampicin (100 μg/ml) and streptomycin (200 μg/ml) was used. For vST detection, xylose lysine deoxycholate agar (XLD) supplemented with rifampicin (100 μg/ml) and nalidixic acid (5 μg/ml) was used. BGA and XLD plates were incubated at 37°C for 24 ± 3 h and 48 ± 3 h, respectively, and colonies enumerated. Representative colonies were tested by slide agglutination (Poly O A-S, Pro-Lab Diagnostics, UK) for confirmation of being *Salmonella*. Both vaccine strains are resistant to rifampicin, and the vSE strain has an additional resistance to streptomycin, while the vST strain has an additional resistance to nalidixic acid. The addition of these antibiotics to media allows positive selection for the vaccine strains and discrimination between the two strains on agar plates. Homogenates were also incubated at 37°C for 20 ± 2 h in order to enrich low levels of vaccines. Where no colonies were detectable from direct plating, enriched samples were plated out for a qualitative result. The limit of detection was 50 cfu/g.

### Statistics

Colonization levels for each vaccination age group were analyzed by *t*-tests (GraphPad Prism). Where vaccine strains were recovered only after enrichment, the median value between 0 and the limit of detection (i.e., 25 cfu/g) was assigned.

## Results

### Vaccine concentrations

Vaccine concentrations were determined in two samples taken from different points along the drinker line in each house on the day of administration. Levels were consistent between each flock and on all occasions. The vSE levels were 3.6 ± 1.5 × 10^7^ cfu/ml, and vST levels were 1 × 10^7^ ± 3.9 × 10^6^ cfu/ml. These levels were as expected and sufficient to meet the prescribed vaccine uptake given expected water consumption levels.

### Vaccine uptake levels

**FIGURE 1:**
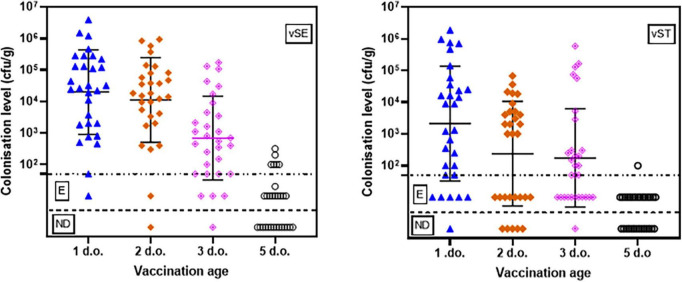
Caecal levels (cfu/g) of vSE and vST in chickens vaccinated at 1-, 2-, 3-, and 5-d.o. Samples were taken 2 days post-vaccination. Detection limit = 50 cfu/g. E–positive only after enrichment; ND, none detected.

Vaccine concentrations were determined two days post-vaccination in caecal and liver samples from 10 chicks randomly selected from each flock. There was a clear trend for higher levels of vaccines in caecal contents the earlier the birds were vaccinated (Figure 1; Table 1 and Supplementary Table 1). In birds vaccinated at 1-d.o. all had detectable levels of vSE, with quantifiable numbers in 29/30 birds. Levels ranged from 50 cfu/g to 10^6^ cfu/g (mean 2.9 × 10^5^ cfu/g). Vaccination at 2- and 3-d.o. both resulted in 29/30 birds with detectable vSE, of which 28/30 and 26/30 resp. were quantifiable (means 1.2 × 10^5^ and 1.8 × 10^4^ cf/g resp). In contrast, in birds vaccinated at 5-d.o. only 16/30 birds had detectable vSE (incl 8/30 with quantifiable levels). Statistical analyses (*t*-tests) revealed there were significant decreases between vaccination at 1- and 5-d.o. (*p* = 0.039), 2- and 3-d.o. (*p* = 0.032), 2- and 5-d.o. (*p* = 0.011) and 3- and 5-d.o. (*p* = 0.027).

**TABLE 1:** Numbers of caecal (C) and liver (L) samples from which vSE and vST were recovered following vaccination of chickens at 1-, 2-, 3-, and 5-d.o (*n* = 30 per age group).

		1do			2do			3do			5do		
		DQ	E	ND	DQ	E	ND	DQ	E	ND	DQ	E	ND
vSE	C	29	1	0	28	1	1	26	3	1	8	8	14
	L	0	12	18	5	25	0	2	9	19	30	0	0
	C and/or L	30		0	30		0	30		0	30		0
vST	C	24	5	1	17	8	5	18	11	1	1	15	14
	L	4	17	9	1	12	17	0	6	24	1	15	14
	C and/or L	30		0	28		2	29		1	19		11

Samples were taken 2 days post-vaccination. C and/or L—numbers of birds where at least one sample type was positive. DQ, directly quantifiable (≥ 50 cfu/g); E, positive only after enrichment; ND, none detected.

For vST, 29/30 birds vaccinated at 1-d.o. had detectable levels, with quantifiable numbers in 24/30 birds. Levels ranged from 50 cfu/g to 10^6^ cfu/g (mean 1.7 × 10^5^ cfu/g). Vaccination at 2-d.o. resulted in 25/30 birds with detectable vSE, of which 17/30 were quantifiable (mean 6.7 × 10^3^ cf/g). With vaccination at 3-d.o. 29/30 birds with detectable vSE, of which 18/30 were quantifiable (mean 3.4 × 10^4^ cf/g). As seen with vSE, there was a notable decrease in vST recovered from birds vaccinated at 5-d.o.: 16/30 birds had detectable levels, with only 1/30 having quantifiable numbers (10^2^ cfu/g). There were significant decreases between vaccination at 1- and 2-d.o. (*p* = 0.034), 1- and 5-d.o. (*p* = 0.027), and 2- and 5-d.o. (*p* = 0.012).

Compared to caecal contents, levels of both vaccines were considerably lower in liver samples (Table 1 and Supplementary Table 1). Where recovered, levels were mostly only detectable after enrichment, and none were detected in many of the birds. The exception to this was for vSE in birds vaccinated at 5-d.o. where all 30 birds had quantifiable levels (mean 2.5 × 10^2^ cfu/g). This was significantly higher than in birds vaccinated at 1-, 2- (both *p* < 0.001) and 3-d.o. (*p* = 0.003).

**FIGURE 2:**
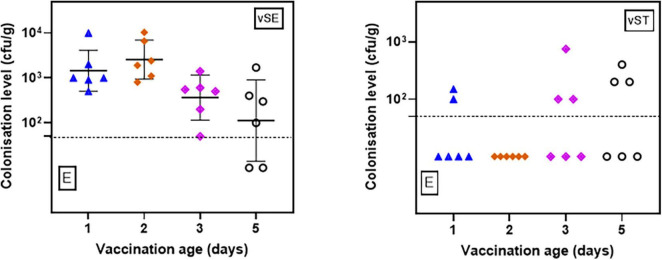
Levels (cfu/g) of vSE and vST in pooled fecal samples from chickens vaccinated at 1-, 2-, 3-, and 5-d.o. Samples were collected 2 days post-vaccination. Detection limit = 50 cfu/g. E–positive only after enrichment.

For the pooled feces, both vaccines were detectable in all samples tested (Figure 2 and Supplementary Table 2). For vSE, all samples had quantifiable levels (range 50 to 10^4^ cfu/g) except 2/6 samples from 5-d.o. vaccinees where enrichment was necessary. Levels of vST were lower and mostly detectable only after enrichment. There were no significant differences (*p* > 0.05) relating to age of vaccination and fecal levels.

## Discussion

It is accepted that vaccination of chickens in rear (both future breeding and laying birds) against *Salmonella* is most successful if a live vaccine is used (Van Immerseel et al., 2005; Desin et al., 2013). Colonization of chicks often happens early in life as a result of hatchery contamination or persistent farm contamination, and leads to high levels of environmental contamination and rapid transmission of pathogens via contaminated litter (Van Immerseel et al., 2005). As inactivated vaccines can’t be administered before six to eight weeks of age, depending on the product, only live vaccines administered via drinking water can provide adequate early protection. Furthermore, oral administration of live *Salmonella* to the newly hatched chicks not only induces an adaptive immune response, but is also able to confer, within 24 h of application, a high degree of resistance against colonization and tissue invasion by other *Salmonella* strains, through a combination of microbiological and innate immunological phenomena (Van Immerseel et al., 2005). From the literature and from earlier studies (Elanco, data on file), we can deduct that colonization of the caeca happens very rapidly after inoculation with the vaccine strain and that caecal levels gradually start to drop after a few days, to disappear around 21 days after vaccination. Berchieri and Barrow (1990) could show that inoculation of chicks within the first 24 h of placement with 10^8^ cfu of an avirulent *Salmonella* strain resulted in 10^8^ organisms found per gram of caecal content a day later; this level was maintained for at least 4 days.

Older studies have previously shown that a specific, locally induced (intestinal) IgA response offers protection against intestinal colonisation by *Salmonella* following challenge (Desmidt et al., 1998). The earlier a vaccine can be administered, the earlier the birds will be protected against exposure to field strains. This is the reason why producers of live vaccines recommend administration as early as 1-day-old. However, the small volumes of water drunk during the first days of a chick’s life, combined with the long drinking water lines often found in poultry houses, sometimes makes it difficult to apply the necessary amount of vaccine over the course of two to four hours, as recommended by most manufacturers. As a consequence, the first dose of vaccine may not be administered on-farm until the birds are several days old as farmers may be concerned about sufficient uptake. In the study described here, vaccination took place over a much longer period than the recommended two to three hours. This was particularly important for birds vaccinated at 1- or 2-days-of-age, where there were concerns birds would not drink sufficient quantities during the vaccination period to enable uptake of appropriate levels of vaccine. A possible downside to prolonged administration is the potential loss of viability of the attenuated vaccine strains. However, despite the long application time, every single bird consumed sufficient volumes to enable detection of live vaccine strains in individual tissue samples (caecum and/or liver), and in all pooled fecal samples collected. Although the producers of live vaccines usually recommend administration of vaccines in drinking water over a short period of time, typically two to four hours, recent data confirm that AviPro™ Salmonella DUO is stable in drinking water for over 12 h (RHConsultancy, UK, personal communication). This helps explain the good results obtained from birds vaccinated at 1- and 2-days-of-age.

Significantly higher levels of vaccine strains in caecal content were found in birds vaccinated earlier in life compared to birds vaccinated slightly later which is perhaps not unexpected as it is known younger birds are more susceptible to colonization by *Salmonella* than older birds (Shivaprasad et al., 2013). In newly-hatched chicks this may be a reflection of an undeveloped gut microbiome as well as an immature innate immune system (Barnes, 1972; Smith et al., 2008). Furthermore, day-of-hatch birds have been shown to be particularly susceptible to bacterial colonization even when there are potentially protective maternally-derived antibodies present (Cawthraw and Newell, 2010). These observations add weight to the idea of administering a live vaccine as early as possible, for reasons of both need and ease of uptake.

The comparatively high levels of vaccine in liver samples in birds vaccinated at 5-d.o. compared to birds vaccinated at 1-d.o. was unexpected, and it is not clear if this finding has any clinical or immunological relevance. Previous data on liver colonization of vaccinated birds were not gathered at such detail, and different vaccination ages of birds were not compared (Elanco, data on file). However, the fact that every bird had at least one positive tissue sample two days after vaccination shows that vaccine uptake was successful although there were differences in liver colonization patterns between the different age groups of birds.

The survivability of live vaccines in the drinking water line depends greatly on water quality and the cleanliness of the line and is easily compromised through unwanted substances or residues as a result of poor hygiene practices. However, the results obtained from this study show that good quality drinking water and/or the use of a stabilizer such as Aviblue supports the survival of AviPro™ Salmonella DUO live vaccine for at least 12 h—long enough to allow all birds, even very young ones, sufficient time to drink enough for successful vaccine uptake. Diligent analysis of the quality of the drinking water and cleanliness of the lines are important considerations for satisfactory vaccination.

The efficacy of the product used in this trial when administered to one-day-old birds via oral gavage has been shown in several studies, for example (Gantois et al., 2006; Eekhout et al., 2018; Huberman et al., 2019), where the vaccinated birds proved to be protected in a challenge experiment. Hence, it was not deemed necessary to perform a challenge experiment, but to focus on the main question instead, which was the successful survival of the vaccine strains in the drinking water line over several hours and the successful uptake of the vaccine by the birds.

In conclusion, the results of this study show that the product AviPro™ Salmonella DUO can be safely and successfully applied to layer pullets as early as 1-day-of-age, despite the necessity to vaccinate over several hours, as long as the quality of the drinking water is carefully monitored.

## Data availability statement

The original contributions presented in this study are included in this article/Supplementary material, further inquiries can be directed to the corresponding author.

## Ethics statement

Ethical approval was not required for the study involving animals in accordance with the local legislation and institutional requirements because the local Ethical Review board (at APHA) were aware of the work and deemed formal review was not required. No experimental procedures were undertaken. The study was a field trial of a vaccine currently licensed for use within the target host (chickens). Normal vaccinal regimes used by the layer industry were followed. Birds would have been vaccinated regardless of this study.

## Author contributions

SC: Conceptualization, Data curation, Formal Analysis, Investigation, Methodology, Supervision, Writing – original draft, Writing – review & editing. AG: Methodology, Supervision, Writing – review & editing. TH: Investigation, Writing – review & editing. IR: Investigation, Writing – review & editing. LC: Investigation, Writing – review & editing. DM-D: Conceptualization, Writing – original draft, Writing – review & editing.
